# Neural Radiance Fields for Fisheye Driving Scenes Using Edge-Aware Integrated Depth Supervision

**DOI:** 10.3390/s24216790

**Published:** 2024-10-22

**Authors:** Jiho Choi, Sang Jun Lee

**Affiliations:** Division of Electronics and Information Engineering, Jeonbuk National University, Jeonju 54896, Republic of Korea; jihochoi@jbnu.ac.kr

**Keywords:** neural radiance field, view synthesis, depth supervision, fisheye camera

## Abstract

Neural radiance fields (NeRF) have become an effective method for encoding scenes into neural representations, allowing for the synthesis of photorealistic views of unseen views from given input images. However, the applicability of traditional NeRF is significantly limited by its assumption that images are captured for object-centric scenes with a pinhole camera. Expanding these boundaries, we focus on driving scenarios using a fisheye camera, which offers the advantage of capturing visual information from a wide field of view. To address the challenges due to the unbounded and distorted characteristics of fisheye images, we propose an edge-aware integration loss function. This approach leverages sparse LiDAR projections and dense depth maps estimated from a learning-based depth model. The proposed algorithm assigns larger weights to neighboring points that have depth values similar to the sensor data. Experiments were conducted on the KITTI-360 and JBNU-Depth360 datasets, which are public and real-world datasets of driving scenarios using fisheye cameras. Experimental results demonstrated that the proposed method is effective in synthesizing novel view images, outperforming existing approaches.

## 1. Introduction

Neural rendering with coordinate-based and implicit functions has evolved as a method for representing 3D objects and scenes from 2D images. Neural volumetric representations have been utilized in various computer vision problems, including novel view synthesis [1], relighting [2], editing [3,4] and pose estimation [5,6], as well as various industrial applications [7,8]. In particular, neural radiance fields (NeRF) [1] have successfully rendered from novel viewpoints with implicit features, resulting in photorealistic image synthesis. NeRF enables us to capture scene geometry with view-dependent emitted radiance with volume density.

NeRF has primarily focused on object-centric scenarios and has conducted experiments using data that specifically revolve around individual objects. Object-centric datasets assume a single, isolated object in a scene, which can lead to challenges when rendering scenes with multiple or complex objects. Moreover, real-world scenes rarely have a single object at the center, highlighting the need for diverse camera trajectories to ensure the scalability of the NeRF. Urban-NeRF [9] aims to render urban environments and enables 3D reconstruction of complex scenes with multiple objects such as buildings. Similarly, S-NeRF [10] extends the applicability of NeRF to realistic and dynamic driving scenarios by proposing street view synthesis using driving datasets. These approaches show ongoing efforts to overcome the limitations of object-centric NeRF algorithms and expand their scope to encompass more diverse and complex scenes.

Previous NeRF algorithms operate predominantly under the assumption of a pinhole camera model. The pinhole model assumes the transmission of light from the real world through a pinhole, forming an inversely oriented image on the camera sensor. However, this approach imposes limitations, primarily the restrictive field of view (FoV) of a pinhole camera, which significantly reduces the capacity to capture comprehensive scenes in a single frame. Furthermore, the idealized pinhole model does not account for real-world camera properties, such as lens distortion. Consequently, NeRF algorithms founded on this model may not provide universally applicable solutions. In response to these limitations, the research introduces a fisheye camera model within the NeRF algorithm. This approach broadens the applicability and enhances the efficiency of scene capture.

Compared to pinhole cameras, fisheye cameras offer the advantage of a wider FoV, typically ranging from $180^{\circ }$ to $220^{\circ }$. A wide FoV camera can capture over $180^{\circ }$ with a single lens and is economically efficient, as it reduces the number of cameras needed to cover an omnidirectional scene. Therefore, fisheye cameras are widely used in autonomous vehicles such as surround monitoring and scene understanding. Previously, various approaches have been explored to utilize fisheye cameras by undistorting the images to address their significant distortion. However, this process results in a loss of image boundary information and degrades overall image quality. To fully exploit the wide FoV, recent efforts have focused on using original fisheye images, and large-scale driving datasets captured by fisheye cameras have been introduced [11,12,13]. In light of this, the proposed method utilizes the original fisheye images without applying distortion correction.

**Figure 1:**
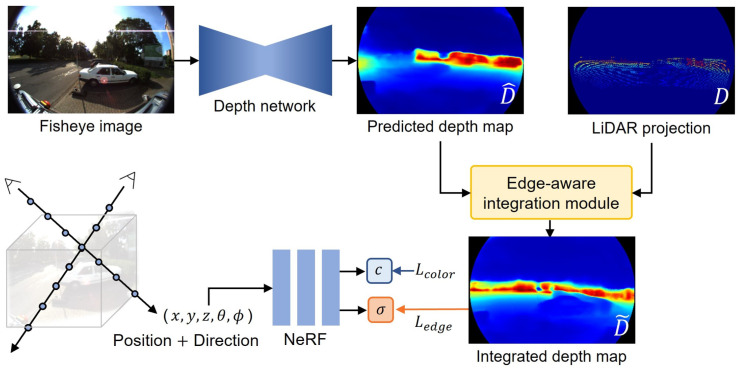
Overview of the proposed pipeline. Given a set of RGB images captured by fisheye camera in driving scenarios, we trained a monocular depth estimation network that outputs the densely predicted depth map $\hat{D}$. Moreover, the LiDAR points are projected to generate a sparse depth map *D*. These two depth priors are employed in the edge-aware integration module. The training of the radiance field is guided by the RGB images and the integrated depth maps $\tilde{D}$, which inform the model regarding ray termination. NeRF takes 5D inputs and is trained using the $L_{color}$ and the proposed $L_{edge}$ loss functions.

In this study, we propose an edge-aware integration loss function designed to minimize the difference in the distribution of rendered rays and integrated depth. The proposed method utilizes accurate depth data obtained from the LiDAR sensor, coupled with estimated dense depth information, which carries inherent uncertainty. Specifically, pixels adjacent to the LiDAR point rely more on sensor data than on the estimated depth. Moreover, we consider the edge component by assigning larger weights to pixels that have similar depth. Figure 1 shows an overview of the proposed method. The effectiveness of the proposed algorithm was evaluated on driving datasets including the KITTI-360 and the custom dataset.

In summary, we propose a NeRF that employs a fisheye camera model aimed at driving scenarios, rather than a conventional object-centric approach. The implementation code is available at https://github.com/ziiho08/Edge-aware-Integrated-Depth-Supervision (accessed on 17 October 2024). The contributions are the following:

- We present a method that utilizes a fisheye camera to synthesize novel views in real-world driving scenarios.
- We propose an edge-aware integration loss function that minimizes the difference between the rendered ray and the integrated depth distribution.
- We demonstrate that the proposed method outperforms other approaches, as shown by the results from the KITTI-360 [13] and JBNU-Depth360 datasets.

## 2. Related Work

### 2.1. Fisheye and Omni-Directional NeRF

NeRF contains the radiance field of a scene in a neural network and generates photorealistic renderings of novel views. Various approaches have been proposed to improve rendering performance in terms of anti-aliasing [14], faster training and inference [15], and generalization [16]. However, most previous methods rely on the pinhole camera model, and research on other camera models remains limited. Recent works have proposed NeRF based on camera models other than the traditional pinhole camera. Omni-NeRF [17] proposed a spherical sampling method for synthesizing novel views using fisheye images, demonstrating scene reconstruction for both synthetic and real fisheye camera datasets. 360FusionNeRF [18] introduced methods to render novel views from $360^{\circ }$ panoramic images in RGB-D, using geometric supervision to enhance rendering accuracy. Moreover, DiCo-NeRF [19] integrated a vision-language model to enhance the performance of novel view synthesis in fisheye driving scenes. These studies demonstrate that NeRF is not limited to a specific camera type but can be effectively applied to various camera models, including fisheye cameras. Notably, the wide FoV camera enable the rendering of broad scenes within a single image.

The SCNeRF [6] approach proposes a method for handling generic camera models by performing self-calibration to optimize NeRF without relying on pose information. This technique enables the rendering of generic camera models, including those with complex non-linear camera distortion coefficients. The study showed experimental results on the fisheye image dataset and demonstrated its applicability to various variants of NeRF. However, they predominantly focus on static fisheye images, suggesting the potential for further exploration in dynamic scenarios. To address this gap, our work presents a method for synthesizing novel views in driving scenarios using fisheye images, thereby expanding the applicability of NeRF algorithms.

### 2.2. NeRF with Depth Supervision

Several studies have successfully performed 3D reconstruction by supervising the NeRF model with additional depth information. NerfingMVS [20] introduces an optimization framework that incorporates adapted learning-based depth priors to regulate the sampling process of NeRF during volume rendering. The experimental results of the indoor ScanNet dataset demonstrate the effectiveness of their approach. Moreover, DS-NeRF [21] leverages sparse 3D point clouds from structure-from-motion (SFM) COLMAP [22] to improve rendering results with fewer training views, while achieving faster training speeds compared to traditional NeRF. Several studies demonstrated promising results in utilizing LiDAR data for novel view synthesis tasks. Urban NeRF [9] utilizes RGB images and asynchronously recorded LiDAR points to enhance the reconstruction of large outdoor scenes. They have demonstrated state-of-the-art 3D surface reconstructions and realistic novel views in their experiments conducted on street view data. S-NeRF [10] takes advantage of sparse LiDAR data from autonomous vehicles to enhance neural representation learning, improving street view synthesis. They simultaneously rendered large-scale background scenes and moving foreground vehicles, and conducted experiments on substantial driving datasets, namely nuScenes [23] and Waymo [24].

Recently, SparseNeRF [25] introduced local depth ranking to enhance performance with limited views by utilizing imprecise depth priors. Additionally, [26] provided a comprehensive review of the use of depth priors in outdoor scenarios for NeRF, evaluating various depth types to evaluate their influence on performance. These methodologies have shown promising results on large driving datasets, underlining their ability to effectively reconstruct urban and street scenes.

## 3. Method

We propose a novel NeRF algorithm that utilizes depth supervision. Synthesizing wide FoV images in driving scenarios is a difficult problem due to the strong distortion and unbounded characteristics. To address these challenges, we introduced dense depth maps inferred by a learning-based model to supplement sparse LiDAR projections. The edge-aware integration loss is a function for effectively employing the sparse and dense depth maps.

### 3.1. Preliminaries

A NeRF encodes scenes by representing them as a color value $c\in {\left[0,1\right]}^{3}$ and density $\sigma \in \left[0,\infty \right)$. This representation is achieved through a multi-layer perceptron (MLP), which serves as an implicit function for modeling the scenes. The NeRF model receives a 3D coordinate $x\in R^{3}$ and a viewing direction $d\in S^{2}$ as inputs and predicts the corresponding density and RGB color values. This process is expressed by the equation: $f\left(x,d\right)=\left(\sigma ,c\right)$, where *f* is the MLP that generates the density and RGB color values. To capture high-frequency details, NeRF applies positional encoding to its input, mapping them to a higher-dimensional space using sinusoidal functions of various frequencies. This encoding process is defined as follows:

(1) $$\gamma_{L}\left(x\right)=\left[sin\left(x\right),cos\left(x\right),...,sin\left(2^{L-1}x\right),cos\left(2^{L-1}x\right)\right],$$

where *L* is a parameter that controls the maximum frequency used in the positional encoding [1]. For rendering a pixel’s intensity, a ray r(t) is cast from the camera’s center in the direction d, with *t* representing the distance. The color value is obtained by integrating the radiance fields from $t_{n}$ to $t_{f}$ as described by the following Equation [1]:

(2) $$\begin{array}{c} \hat{c}\left(r\right)=\int_{t_{n}}^{t_{f}}T\left(t\right)\sigma \left(r\left(t\right)\right)c\left(r\left(t\right),d\right)dt,\\ where\;T\left(t\right)=exp(-\int_{t_{n}}^{t_{f}}\sigma \left(r\left(s\right)\right)ds). \end{array}$$

In Equation (2), σ and c are the density and color values predicted by the function *f*, and T(t) represents the transmittance along the ray up to the distance *t*. The ray termination distribution is denoted as h(t) and can be calculated as h(t)=T(t)σ(t). The NeRF model is trained by minimizing the color difference between the predicted and actual values [1], as denoted in the loss function:

(3) $$L_{color}=\sum_{r\in R_{i}}{∥\hat{c}\left(r\right)-c\left(r\right)∥}^{2},$$

where $R_{i}$ represents the set of rays for the *i*-th image.

### 3.2. Depth Supervision

The proposed method employs sparse LiDAR projections and dense depth predictions. The LiDAR projections are denoted and $D=\left\{D_{i}\in R^{H\times W}|i=0,\cdots ,N-1\right\}$, where *H* and *W* represent the height and width, and *N* is the number of images. Although the LiDAR projection provides accurate depth information, it suffers from a sparsity of valid points. To address this limitation, we incorporate depth predictions, denoted as $\hat{D}=\left\{{\hat{D}}_{i}\in R^{H\times W}|i=0,\cdots ,N-1\right\}$, inferred from a learning-based depth network. To simplify the equation, we will omit the subscript *i* in the following explanations.

Recent advancements in supervised depth estimation algorithms have shown promising results in multiple datasets including NYU-Depth-v2 [27] and KITTI [28]. Motivated by the success, we aim to improve the performance of a NeRF model by incorporating depth information inferred from a depth estimation model. We trained a monocular depth estimation model [29], which takes an image to estimate a dense depth map $\hat{D}$. While the predicted depth map contains the knowledge of terminating the rays, it contains uncertainty for predicting the accurate depth information. To address the sparsity of the *D* and uncertainty of $\hat{D}$, we integrated the sparse and dense depth maps to improve the reliability of the depth prior.

### 3.3. Edge-Aware Integration Loss

**Figure 2:**
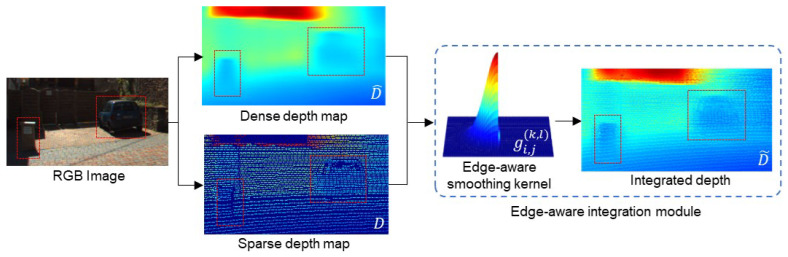
Process of integrating the sparse LiDAR projection and dense estimated depth map. We propose the edge-aware integration loss function to optimize the NeRF model with depth supervision, as detailed in (4). The proposed method guides the NeRF model using depth priors from the scene by minimizing the difference between the distributions of ray termination from the model and the given depth information. This depth is determined using the edge-aware smoothing kernel, which takes advantage of both depth priors. Moreover, it assigns a larger weight to adjacent points that are consistent with depth values.

In autonomous driving scenarios using fisheye images, novel view synthesis presents unique challenges due to unbounded and distorted characteristics. To address these, we leverage the depth information derived from LiDAR points in the driving datasets as shown in Figure 2. This accurate depth guides the NeRF model in terms of ray termination. Our objective is to minimize the difference between the rendered ray distribution $h_{k}$ and integrated depth ${\tilde{d}}_{i,j}$, and it is referred to as the edge-aware integration loss:

(4) $$L_{edge}=\sum_{i,j}\sum_{k}\;log\;h_{k}\;exp\left(-\frac{{\left(t_{k}-{\tilde{d}}_{i,j}\right)}^{2}}{2{\hat{\sigma }}^{2}}\right)\Delta t_{k},$$

where $h_{k}$ denotes the termination distribution of the ray, $\Delta t_{k}$ represents the ray step, and $\hat{\sigma }$ is the standard deviation of integrated depth map $\tilde{D}$. The components of $\tilde{D}$ at the pixel (i,j) is indicated as ${\tilde{d}}_{i,j}$, and it can be computed as

(5) $$\begin{array}{c} {\tilde{d}}_{i,j}=\eta_{i,j}^{-1}\sum_{k,l}g_{i,j}^{\left(k,l\right)}\left(\hat{D}\right)\left[\alpha \cdot d_{i+k,j+l}\cdot 1\left(d_{i+k,j+l}>0\right)\;\right.\\ \left.+\left(1-\alpha \right)\cdot {\hat{d}}_{i+k,j+l}\cdot 1\left(d_{i+k,j+l}=0\right)\right]. \end{array}$$

We obtain ${\tilde{d}}_{i,j}$ by integrating a sparse LiDAR projection map with a dense prediction map using the explicit function, and $\eta_{ij}$ is the normalization factor defined as

(6) $$\begin{array}{c} \eta_{i,j}=\sum_{k,l}g_{i,j}^{\left(k,l\right)}\left(\hat{D}\right)\left[\alpha \cdot 1(d_{i+k,j+l}>0)\right.\\ \left.+\left(1-\alpha \right)\cdot 1\left(d_{i+k,j+l}=0\right)\right], \end{array}$$

where α is a hyper-parameter that controls the effect of sparse and dense depth maps, and it was set to 0.7. In experiments, the kernel size was set to 9, and the indexes *k* and *l* are integers in $\left[-4,4\right]$.

In the edge-aware integration module, as described in (5), we factor in the locations of the neighbors of the pixel (i,j) within the kernel to integrate two distinct depth maps. The dense depth ${\hat{d}}_{i+k,j+l}$ surrounding the LiDAR point $d_{i,j}$ is refined using neighboring sensor data. This refinement involves assigning a larger weight to pixels closer to the LiDAR information. Importantly, pixel values outside an object’s edge differ significantly from the depth values within its boundary. To address this, we consider the depth differences between LiDAR and inferred depth to resolve the depth ambiguity. We term these procedures the edge-aware smoothing kernel, denoted as

(7) $$g_{i,j}^{\left(k,l\right)}\left(\hat{D}\right)=exp\left(-\frac{k^{2}+l^{2}}{2\sigma_{s}^{2}}\right)\cdot exp\left(-\frac{{\left({\hat{d}}_{i,j}-{\hat{d}}_{i+k,j+l}\right)}^{2}}{2\sigma_{d}^{2}}\right),$$

where $\sigma_{s}$ and $\sigma_{d}$ represent the standard deviation of spatial and depth spaces; they were set to 3 and 1, respectively. This smoothing kernel helps to preserve edge components while ensuring the diffusion of accurate depth data from the sensor.

The proposed approach aims to optimize the NeRF for fisheye driving scenarios. We achieve this by employing the proposed edge-aware integration loss function, which leverages two different depth priors. The total loss function for NeRF is $L_{total}=L_{color}+\lambda L_{edge}$, and λ determines the balance between the loss functions.

## 4. Experimental Results

We evaluated the proposed method using the KITTI-360 [13] and JBNU-Depth360 datasets, which include fisheye driving scenes in both outdoor and parking lot environments. In these experiments, we compared the method with previous NeRF algorithms, including nerfacto [30], UrbanNeRF [9], and DS-NeRF [21]. In addition, ablation studies were conducted to demonstrate the effectiveness of the edge-aware integration module and to explore the impact of different depth types, including the LiDAR projection and the estimated depth from a depth network.

### 4.1. Datasets

- KITTI-360.

This dataset includes 320 k images and 100 k laser scans collected from driving scenarios in the suburbs of Karlsruhe, Germany. It contains RGB images captured with $180^{\circ }$ FoV fisheye cameras and 3D point clouds acquired from an HDL-64E Velodyne LiDAR sensor. The fisheye images have a resolution of 1400×1400, and the point clouds were projected onto the 2D image plane to generate sparse ground-truth depth maps. The dataset encompasses nine different driving scenarios and we used 150 images from each scenario to train the NeRF model. In the experiments, we utilized known intrinsic parameters, and camera poses were determined using the COLMAP algorithm.

- JBNU-Depth360.

This custom dataset was collected in underground parking lot environments using a Jackal UGV mobile robot equipped with a NVIDIA Jetson AGX Xavier, $183^{\circ }$ FoV fisheye cameras, and a $90^{\circ }$ FoV Ouster LiDAR sensor. The dataset includes 4221 RGB images with a resolution of 1080×1920, and the 3D LiDAR points were projected onto the image plane to generate sparse ground-truth depth maps. It consists of six driving scenarios, and we utilized approximately 126 images for the training of a NeRF model in each scenario. The training images were selected based on time intervals, and the relative camera poses were computed by using the COLMAP.

- Evaluation metric.

We report the quantitative comparison results using metrics such as the peak signal-to-noise ratio (PSNR), the structural similarity index measure (SSIM), and the learned perceptual image patch similarity (LPIPS) in the output synthesized images of novel views.

### 4.2. Results on the KITTI-360 Dataset

We compare the method with nerfacto [30], UrbanNeRF [9] and DS-NeRF [21] on the KITTI-360 datasets. Table 1 shows the image synthesis metrics achieved by the proposed method on KITTI-360 dataset. We compare the proposed method with the base pipeline nerfacto, as well as the loss functions of DS-NeRF and UrbanNeRF. We tested these previous depth-based methods using the LiDAR projection as the ground truth. In most driving scenarios, our approach outperforms the previous depth supervision methods across all metrics. Specifically, the proposed method achieves average PSNR, SSIM, and LPIPS of 12.427, 0.535, and 0.574, respectively. The findings indicate that optimizing the NeRF model with depth supervision improves geometry accuracy by better understanding the ray termination distribution. This optimization is effective in reproducing the photorealistic fisheye images.

**Figure 3:**
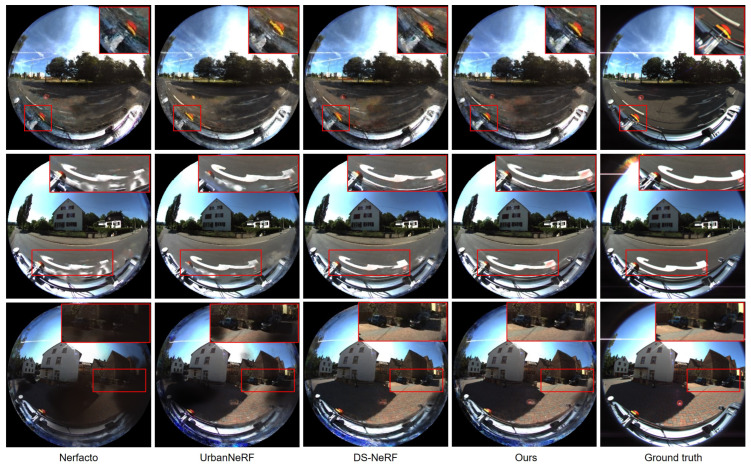
View synthesis on KITTI-360 dataset. The proposed method has demonstrated improved photorealistic results, as highlighted in the red boxes.

Figure 3 further highlights the effectiveness of the proposed approach through qualitative results. As shown in the first row, the proposed method’s radiance representation avoids the blurry and floating artifacts commonly observed in other methods, particularly for objects near the camera. Additionally, it consistently produces sharper results for devices mounted on the vehicle. By optimizing NeRF with depth priors, the approach effectively reduces these artifacts, resulting in more realistic color synthesis and appearance. The results clearly depict the road surface without the blurring typically seen in previous methods, and the proposed method successfully generates images from novel views, outperforming earlier approaches. These findings demonstrate that the integrated depth information considerably enhances the quality of the novel view synthesis task.

### 4.3. Results on the JBNU-Depth360 Dataset

The method was also evaluated on a custom dataset collected from parking lot driving scenarios. The quantitative results of the view synthesis are presented in Table 2. Our proposed method has demonstrated significant improvements in both PSNR and SSIM metrics, achieving averages of 20.511 and 0.764, respectively. Furthermore, there was a notable reduction in LPIPS, which decreased from 0.541 of DS-NeRF to 0.529. This suggests that images rendered by the proposed method are more realistic compared to those synthesized by previous depth supervision approaches. We demonstrated the method in both public datasets and real-world data, including both indoor and outdoor environments.

**Figure 4:**
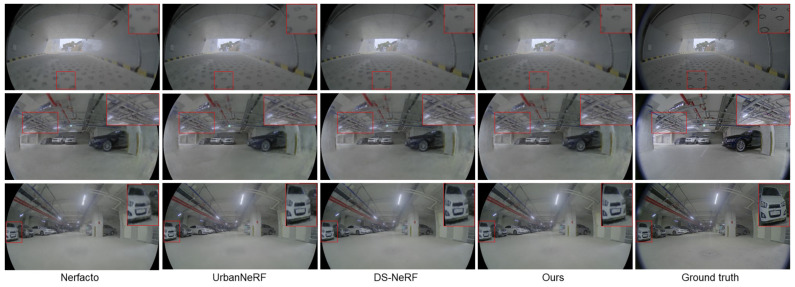
View synthesis on JBNU-Depth360. We have highlighted the details of the synthesized image with a red box.

Figure 4 presents a qualitative comparison of novel view synthesis using the JBNU-Depth360 dataset. In the first row, it is shown that previous methods often produce blurry results, particularly in floor details. In contrast, the proposed method effectively captures these details, reproducing them with higher fidelity to the ground truth data. The results in the second row further demonstrate that the proposed approach preserves even intricate structures. These findings suggest that integrating accurate sensor depth with densely inferred depth information leads to a more realistic synthesis.

### 4.4. Ablation Study

- Depth type comparison.

We investigate the impact of different depth types in Table 3. Both LiDAR projections and estimated depth maps provide advantages when optimizing the NeRF. Despite having fewer valid pixels, sparse LiDAR projections show similar PSNR and higher SSIM than the prior estimated depth. This result indicates that the accurate sensor data guided the NeRF model in learning the ray termination distribution and enhancing scene rendering. However, the dense depth map from the depth estimation model outperforms the view synthesis results from LiDAR data in terms of LPIPS. This implies that it provides more comprehensive perceptual information to the NeRF, even with some inherent uncertainties. Consequently, our approach demonstrated superior performance across all metrics, suggesting that the method of depth integration contributed significantly to optimizing the NeRF for realistic scene rendering.

- Depth integration method comparison.

**Figure 5:**
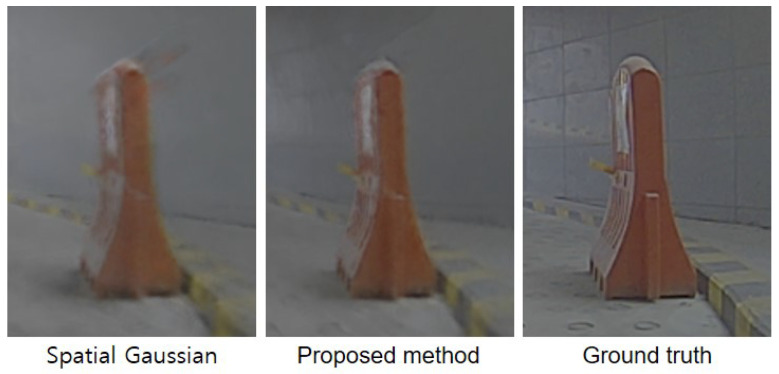
Qualitative results of an ablation study on edge-aware integrated function. Compared to the spatial Gaussian function, the proposed approach better preserves the object’s edge information, resulting in a more realistic representation in the synthetic image.

The edge-aware integration method is proposed to optimally leverage the sparse LiDAR data and prior knowledge of the monocular depth estimation model. In Table 4, we present the results of an ablation study that compares the spatial Gaussian function and the proposed integration method. Across all scenarios, the proposed approach consistently outperformed previous methods, achieving LPIPS of 0.574 and 0.529 for the KITTI-360 and JBNU-Depth360 datasets, respectively. These results indicate that considering the actual depth range is important when combining depth information to avoid depth differences, such as background and vehicles in the scene. Figure 5 shows the view synthesis results obtained from both datasets. We demonstrate that our approach helps preserve edge details by being aware of both spatial and depth range information, which helps NeRF generate realistic images.

## 5. Conclusions

In this work, we introduce a novel approach for synthesizing driving scenarios by leveraging the depth information. The NeRF’s performance significantly degrades when dealing with fisheye-driving images due to the challenges of optimizing for their unbounded and distorted characteristics. It is important to note that driving data presents additional difficulties as it deviates from conventional object-centric datasets. To address these challenges, we propose the edge-aware integration loss that minimizes the discrepancy between the ray termination and integration of LiDAR data with densely estimated depth distribution. We evaluated the proposed method on the KITTI-360 and real-world JBNU-Depth360 datasets, achieving improvements of 0.438 in PSNR and 0.027 in LPIPs, respectively. It demonstrates that the method outperforms previous approaches in rendering novel views. However, the model performs poorly in cases of large rotations of the vehicle, where the number of scenes captured by the camera is relatively small, indicating the need for improvements in few-shot scenarios. The proposed model was validated on a limited dataset due to the scarcity of publicly available fisheye driving datasets. Future research will require the collection of fisheye camera datasets in more diverse driving scenarios. We hope that our work will inspire further research in fisheye rendering, and contribute to the advancement of autonomous driving tasks.

## Figures and Tables

**Table 1:** Quantitative results on the KITTI-360 dataset. Higher is better for PSNR and SSIM and lower is better for LPIPS.

Method	PSNR↑				SSIM↑				LPIPS↓			
	Drive_0002	Drive_0007	Drive_0009	Average	Drive_0002	Drive_0007	Drive_0009	Average	Drive_0002	Drive_0007	Drive_0009	Average
Nerfacto [30]	13.466	13.699	13.650	13.024	0.580	0.583	0.627	0.537	0.532	0.607	0.439	0.583
UrbanNeRF [9]	13.645	13.765	13.997	13.099	0.579	0.580	0.629	0.539	0.551	0.603	0.436	0.590
DS-NeRF [21]	13.673	14.262	14.023	13.240	**0.580**	0.577	0.631	0.538	0.532	0.603	0.439	0.584
Ours	**14.180**	**14.920**	**14.102**	**13.678**	0.577	**0.594**	**0.641**	**0.548**	**0.521**	**0.594**	**0.641**	**0.557**

**Table 2:** Quantitative results on the JBNU-Depth360 dataset. Higher is better for PSNR and SSIM and lower is better for LPIPS.

Method	PSNR↑					SSIM↑					LPIPS↓				
	Drive_2	Drive_3	Drive_4	Drive_5	Average	Drive_2	Drive_3	Drive_4	Drive_5	Average	Drive_2	Drive_3	Drive_4	Drive_5	Average
Nerfacto [30]	20.102	19.811	20.155	19.002	20.388	0.763	0.737	0.754	0.749	0.763	0.561	0.544	0.524	0.565	0.542
UrbanNeRF [9]	20.130	19.822	20.164	19.170	20.452	**0.764**	0.737	0.754	0.750	0.763	0.563	0.539	0.521	0.561	0.540
DS-NeRF [21]	20.157	19.819	20.341	19.071	20.468	0.763	0.738	0.754	0.750	0.763	0.564	0.540	0.521	0.568	0.541
Ours	**20.177**	**20.028**	**20.356**	**19.214**	**20.511**	0.763	**0.739**	**0.755**	**0.755**	**0.764**	**0.553**	**0.526**	**0.509**	**0.556**	**0.529**

**Table 3:** Effect of depth type including LiDAR and estimated depth. We compare different types of depth information using the KITTI-360 and JBNU-Depth360 datasets.

	Depth Type	PSNR↑	SSIM↑	LPIPS↓
KITTI-360	LiDAR projection	13.240	0.538	0.584
	Estimated depth	13.555	0.539	0.578
	Integrated depth	**13.678**	**0.548**	**0.557**
JBNU-Depth360	LiDAR projection	20.468	0.763	0.541
	Estimated depth	20.401	0.761	0.542
	Integrated depth	**20.511**	**0.764**	**0.529**

**Table 4:** Integration method comparison. We conducted an ablation study on the integration method in terms of novel view synthesis using the KITTI-360 and JBNU-Depth360 datasets.

	Method	PSNR↑	SSIM↑	LPIPS↓
KITTI-360	Spatial Gaussian	13.502	0.533	0.592
	Proposed method	**13.678**	**0.548**	**0.557**
JBNU-Depth360	Spatial Gaussian	19.899	0.753	0.549
	Proposed method	**20.511**	**0.764**	**0.529**

## Data Availability

The KITTI-360 dataset is publicly available online, and it can be found at https://www.cvlibs.net/datasets/KITTI-360, accessed on 17 October 2024. The JBNU-Depth360 dataset is available at https://github.com/EunjinSon1/JBNU-Depth360, accessed on 17 October 2024.
